# Expert opinions from the 2024 closed door round table discussion on improving stroke prehospital care globally

**DOI:** 10.1111/cns.14746

**Published:** 2024-05-10

**Authors:** Renyu Liu, Jing Zhao, Jeyaraj Durai Pandian, Gary A Ford, Qiuhong Ji, Siju V. Abraham, Xunming Ji, Anthony Rudd

**Affiliations:** ^1^ Department of Anesthesiology and Critical Care Perelman School of Medicine at the University of Pennsylvania Philadelphia Pennsylvania USA; ^2^ Department of Neurology Perelman School of Medicine at the University of Pennsylvania Philadelphia Pennsylvania USA; ^3^ Department of Neurology Minhang Hospital Affiliated to Fudan University Shanghai China; ^4^ Department of Neurology Christian Medical College Vellore India; ^5^ Radcliffe Department of Medicine University of Oxford, UK Oxford UK; ^6^ Department of Neurology Nantong University School of Medicine Nantong China; ^7^ Department of Emergency Medicine Jubilee Mission Hospital, Medical College & Research Institute Kerala India; ^8^ Department of Neurosurgery Xuanwu Hospital, Capital University Beijing China; ^9^ Stroke Research Group and Division for Health & Social Care Research King's College London London UK

Stroke is a leading cause of death and disability worldwide, with a significant burden in low‐ and middle‐income countries (LMICs).^1^ Timely access to stroke care is crucial for improving patient outcomes, highlighting the importance of effective prehospital care systems. In recognition of this need, the World Stroke Organization Taskforce for Prehospital Care (WSOTPC) convened a closed‐door round table discussion in Nantong, China on March 3, 2024 followed by continued online discussions via emails to consolidate some key perspectives. The discussion was moderated by Dr. Renyu Liu (Co‐Chair, WSOTPC, University of Pennsylvania, USA), and included participation of Dr. Jeyaraj Durai Pandian (President‐elect, WSO, Christian Medical College, India), Dr. Jing Zhao (Co‐Chair, WSOTPC, Fudan University, China), Dr. Gary Ford (Creator of Face Arm Speech Test (FAST), Oxford University, UK), Dr. Xunming Ji (Director, Millions of Disability Reduction Project for Stroke in China, Capital University, China), Dr. Anthony Rudd (President, Coalition of Special Taskforces for Stroke, CSTS; Kings College, London UK), and Dr. Qiuhong Ji (Local Hosting Chair, Nantong University, China; Figure 1: Group photos of the round‐table participants).

**FIGURE 1:**
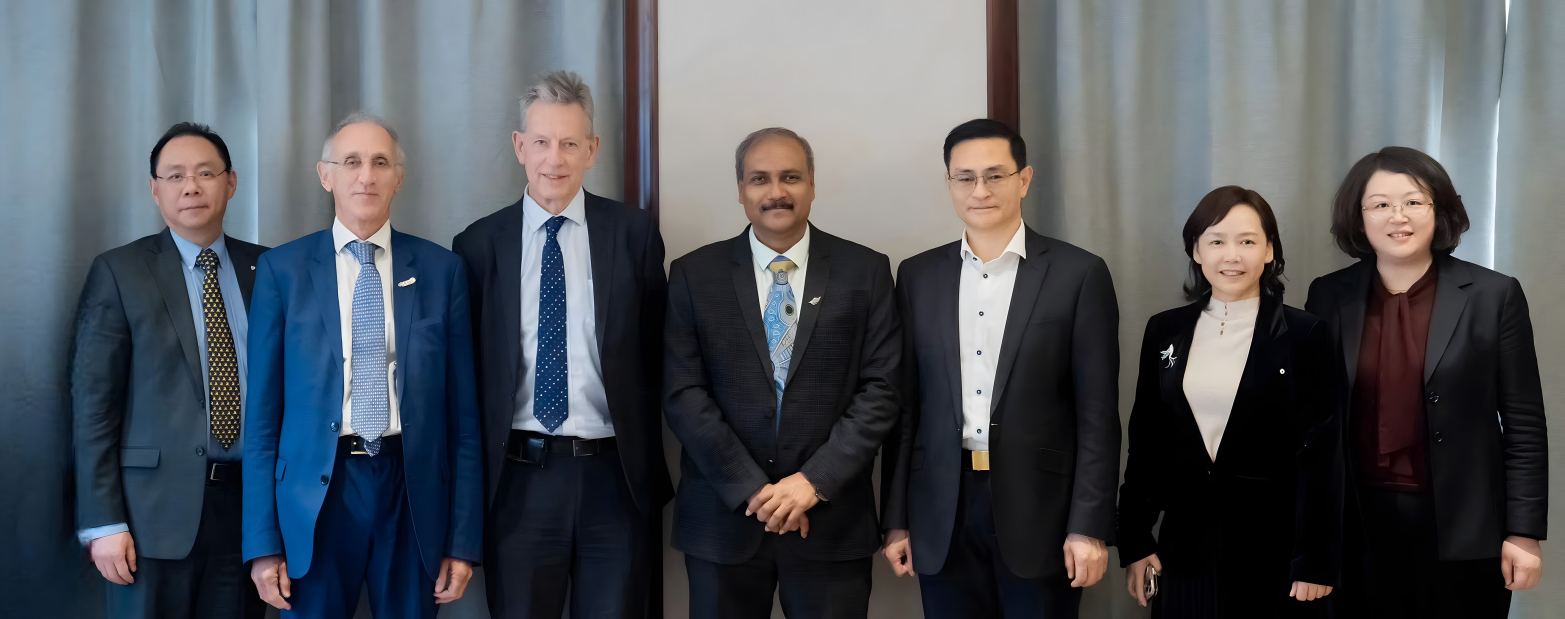
Group photo for the closed‐door round table discussion on the stroke prehospital care. From left to right: Renyu Liu, Anthony Rudd, Gary Ford, Jeyaraj Durai Pandian; Xunming Ji, Jing Zhao, Qiuhong Ji. Dr. Siju V Abraham participated post‐round table discussion and paper editing (not in the photo).

The following key expert opinions and agreements emerged from the round table:

1. *Prioritizing rapid recognition and transfer*: Rapid recognition and immediate action to transfer to hospital via an emergency medical system (EMS) is the most effective intervention. To improve stroke awareness is a top priority to decrease stroke pre‐hospital delay.^2^ Educational tools and public awareness campaigns with culture tailored methodology like Stroke 120 implemented in China with outstanding effectiveness^3^, ^4^, ^5^ are vital and should be further developed. Some of other examples are Stroke 911^6^ and Stroke 112.^7^, ^8^
2. *Cost‐effectiveness*: With the rapid development in hospital care systems, improving stroke prehospital care is likely to be the most cost effective way of reducing stroke burden, morbidity and disability.
3. *Sharing best practices*: Sharing best practices and utilizing case studies were seen as valuable resources especially for LMIC implementation. The WSOTPC plans to develop case studies demonstrating effective interventions to improve stroke prehospital care that could be suitable for implementation in many countries and regions.
4. *Prehospital stroke care specific guidelines*: The development of specific guidelines/recommendations for stroke prehospital care was deemed essential. These guidelines/recommendations should encompass training protocols, patient transfer procedures, tele‐stroke implementation, and innovative stroke detection methods. Adaptation and suitability to the resource constraints of LMICs needs to be considered.
5. *Focus on onset to door time* (*ODT*): The quality of the stroke prehospital care should be considered to be included as part of the stroke center certification process. ODT (onset to door time) is ultimately the measurement that will determine the effectiveness of reperfusion treatment rather than DNT (door to needle time). It should be recommended as a quality evaluation indicator for stroke care.
6. *Prehospital stroke care focused research*: High quality clinical trials are needed for these areas of stroke prehospital care. The team will collaborate with international experts to develop a series of reviews along with case studies for publication identifying the evidence and potential solutions to be addressed in stroke prehospital care, especially in LMICs. While there are recent systemic reviews about stroke prehospital care,^9^, ^10^ these mainly focused on well‐developed nations.
7. *Funding and Innovation*: Stroke prehospital care is under‐developed, and underfunded.^11^ The taskforce should expand its efforts to improve funding, innovation and research in this area.
8. *Sustained collaboration*: An annual meeting and expert round table discussion should occur to maintain the momentum to improve stroke prehospital care by identifying issues and potential solutions across the world with a focus on LMICs. Webinars dedicated to stroke prehospital care two to three times a year should be planned in association with the WSO.
9. *Expansion of taskforces*: The need to expand the taskforces for prehospital care to include more experts especially these from LMICs or these who are interested in improving stroke care in LMICs was acknowledged to ensure a more representative and inclusive approach to global stroke prehospital care improvement.

During the group discussion, participants also discussed the importance of prompt hospital transfer for patient's ineligible for thrombolysis or thrombectomy, and in areas lacking such services.

Discussion also addressed the need for prehospital services to effectively manage minor strokes and transient ischemic attacks, thus preventing major strokes.

There has been rapid development of mobile stroke unit in many countries including in China. However, it was acknowledged that many MSU are usually based in, or close to comprehensive stroke centers in city centers where the population is predominantly young people with a low incidence of stroke and also areas where patients will anyway be taken directly to a comprehensive stroke center. Concerns were raised about the accessibility of MSU for elderly individuals who are mostly living in suburbs and rural areas. Future planning should consider such factors when developing policy.

An important outcome was the productive dialogue between Dr. Pandian, WSO president‐elect, and Dr. Ji (Xunming), director of the Chinese Stroke Center Association. They discussed the potential for a joint certification program between WSO and the Chinese Stroke Center Association, with Dr. Pandian proposing the introduction of the WSO‐endorsed Angel Program. Dr. Pandian expressed a desire for increased Chinese expert participation in WSO activities, indicating mutual interest in enhancing collaboration. All the participants felt that the round table discussion was productive.

In summary, the First Closed‐Door Round Table Discussion on Stroke Prehospital Care effectively brought together leading experts to chart a path forward to improve stroke prehospital care. Consensus focused on culturally sensitive public education, LMIC‐tailored guidelines and prioritizing onset‐to‐door time data. The discussion also highlighted the need for increased prehospital care focused research, funding, and collaboration. This round table discussion summary provides valuable perspectives to inform future strategies for optimizing stroke prehospital care delivery and improving patient outcomes globally, with a particular emphasis on addressing the stroke burden in LMICs.

## CONFLICT OF INTEREST STATEMENT

The authors declare that they have no competing interests.

## Data Availability

Data are available upon reasonable request.

## References

[1] Feigin VL , Brainin M , Norrving B , et al. World stroke Organization (WSO): global stroke fact sheet 2022. Int J Stroke. 2022;17(1):18‐29. 34986727 10.1177/17474930211065917

[2] Rudd AG , Zhao J , Ford G , et al. Results of an international survey on the status of prehospital care. Int J Stroke. 2023;18(9):1084‐1091. 37154607 10.1177/17474930231177204 PMC10614170

[3] Yuan J , Li M , Liu Y , et al. Analysis of time to the hospital and ambulance use following a stroke community education intervention in China. JAMA Netw Open. 2022;5(5):e2212674. 35579896 10.1001/jamanetworkopen.2022.12674 PMC9115614

[4] Zhao J , Li X , Liu X , et al. Changing the strategy and culture of stroke awareness education in China: implementing stroke 1‐2‐0. Stroke Vasc Neurol. 2020;5(4):374‐380. 32350059 10.1136/svn-2019-000324 PMC7804060

[5] Zhao J , Liu R . Stroke 1‐2‐0: a rapid response programme for stroke in China. Lancet Neurol. 2017;16(1):27‐28. 10.1016/S1474-4422(16)30283-6 PMC5578829 28029517

[6] Liu R , Zhao J , Li X , Messe S , Fisher M , Rudd A . To use stroke 911 to improve stroke awareness for countries where 911 is used as an emergency phone number. CNS Neurosci Ther. 2022;28(10):1473‐1475. 35924380 10.1111/cns.13931 PMC9437232

[7] Melifonwu R , Onwuekwe I , Zhao J , Liu R . Prehospital stroke care in Africa: the reality and potential solutions. CNS Neurosci Ther. 2023;29(1):5‐7. 10.1111/cns.14005 PMC9804051 36317707

[8] Zhao J , Eckenhoff MF , Sun WZ , Liu R . Stroke 112: a universal stroke awareness program to reduce language and response barriers. Stroke. 2018;49(7):1766‐1769. 29925649 10.1161/STROKEAHA.118.021729 PMC6034704

[9] Richards CT , Oostema JA , Chapman SN , et al. Prehospital stroke care part 2: on‐scene evaluation and management by emergency medical services practitioners. Stroke. 2023;54(5):1416‐1425. 36866672 10.1161/STROKEAHA.123.039792 PMC10133016

[10] Zachrison KS , Nielsen VM , de la Ossa NP , et al. Prehospital stroke care part 1: emergency medical services and the stroke systems of care. Stroke. 2023;54(4):1138‐1147. 36444720 10.1161/STROKEAHA.122.039586 PMC11050637

[11] Zhao J , Ren L , Abraham SV , et al. The stroke prehospital delay summary statement: a global battlefield. Transl Perioper Pain Med. 2019;6(1):20‐26.

